# Integrative Transcriptomic and Metabolomic Analyses Provide Insights into the Regulatory Basis of *Streptomyces pactum* Act12-Associated Root Development in Wheat

**DOI:** 10.3390/plants15101443

**Published:** 2026-05-08

**Authors:** Jinhui Zhang, Hongwei Wen, Yuzhi Wang, Zeyu Wang, Xingwei Zheng, Hao Shan, Bin Yang

**Affiliations:** Key Laboratory of Sustainable Dryland Agriculture of Shanxi Province, Institute of Wheat Research, Shanxi Agricultural University, Linfen 041000, China; zhangjinhui@sxau.edu.cn (J.Z.); sxnkywhw@163.com (H.W.); y20233112@163.com (Z.W.);

**Keywords:** PGPR, root, *Streptomyces pactum*, transcriptomic, metabolomic

## Abstract

Plant growth-promoting rhizobacteria (PGPR) provide critical ecological value in sustainable agriculture by enhancing plant growth and stress tolerance through improved nutrient acquisition and increased environmental adaptability. As a versatile genus of PGPR, *Streptomyces* shows great potential for promoting plant growth. However, the molecular mechanisms by which *Streptomyces* regulates plant root development remain largely unclear. In this study, we explored the molecular basis associated with wheat root developmental responses to *Streptomyces pactum* Act12 using pot experiments combined with multi-omics approaches. The pot experiment demonstrated that Act12 treatment significantly increased wheat biomass and enhanced total root length (44.6%), root surface area (73.3%), root diameter (34.0%), and root tip number (66.6%). Integrated transcriptomic and metabolomic analyses suggested that Act12 treatment was associated with altered auxin signaling and coordinated changes in carbohydrate metabolism, the TCA cycle, and sterol biosynthesis. These multi-omics signatures provide hypotheses for how Act12 may contribute to root developmental regulation in wheat.

## 1. Introduction

The root system serves as the primary organ responsible for substance exchange and signal communication between plants and their surrounding environment. Root developmental status directly affects the efficiency of soil resource utilization, which in turn influences plant growth, stress tolerance, and ultimately yield formation [1]. Studies have shown that root architecture traits, including total root length, root surface area, and root tip number, are significantly positively correlated with aboveground plant biomass [2]. A well-developed root system provides robust mechanical support for shoot and leaf growth, while modulating aboveground growth, development, and stress tolerance via signal transduction [3,4]. Against the backdrop of a growing global population, shrinking arable land, and increasingly frequent extreme weather events, improving crop productivity by green and sustainable strategies to modulate root system development has become a major research focus and pressing demand in modern agriculture.

Plant growth-promoting rhizobacteria (PGPR) are beneficial microorganisms that colonize the rhizosphere and root surfaces. They promote plant growth, enhance nutrient uptake, and improve stress tolerance through both direct and indirect mechanisms [5]. Studies have shown that PGPR produce plant hormones such as auxin (IAA), cytokinin (CTK), and gibberellin (GA) to regulate plant cell division and elongation, thereby encouraging root development [6,7,8]. Moreover, some PGPR promote plant growth and increase biomass by emitting volatile compounds. For instance, the volatile organic compound N, N-dimethyl-hexadecylamine (DMHDA) has been reported to stimulate primary root elongation in *Arabidopsis thaliana* [9]. Certain PGPR can also solubilize insoluble mineral nutrients like phosphorus, potassium, and iron in the soil by secreting organic acids, phosphatases, and other compounds, which improves nutrient availability and supports plant growth [10,11,12]. Additionally, PGPR can suppress soil-borne pathogens by secreting antibiotics, chitinase, and other bioactive substances, thereby reducing root disease incidence [13]. They can also trigger systemic resistance in plants, boosting their tolerance to various abiotic stresses [14]. Thanks to these versatile traits, PGPR are widely used in agriculture, promoting sustainable and eco-friendly farming practices [5,15].

*Streptomyces*, a versatile genus of PGPR, has been widely applied in agricultural production. Previous studies have shown that the abundance of *Streptomyces* in the plant rhizosphere is significantly positively correlated with root biomass [16], highlighting its strong plant growth-promoting effects. It has been demonstrated that *Streptomyces* can regulate root architecture by secreting hormones such as IAA [17] and produce secondary metabolites, including pteridic acids, to promote root development [18]. Furthermore, *Streptomyces* improves plant uptake efficiency of mineral nutrients such as nitrogen, phosphorus, and potassium [19,20]. They also enhance plant stress tolerance by inducing systemic resistance via the secretion of compounds with antibacterial and insecticidal activities [21,22]. Collectively, these findings underscore the considerable potential of *Streptomyces* in promoting root development, enhancing nutrient acquisition, and increasing stress tolerance in plants.

However, current studies have mainly focused on the phenotypic and functional characterization of growth-promoting traits induced by *Streptomyces*, including enhanced biomass, improved root architecture, and the identification of key activities such as IAA production, phosphate solubilization, and siderophore production [17,18,22]. The molecular mechanisms underlying how *Streptomyces* activates specific signaling pathways in plant roots and further drives metabolic network remodeling remain largely unexplored. This is particularly evident in gramineous crops such as wheat, where root development is tightly coupled with carbon allocation, energy metabolism, and membrane lipid homeostasis [1,23]. However, the systematic responses of these processes at the transcriptional regulatory and metabolic accumulation levels following *streptomycete* inoculation remain poorly understood. It is therefore essential to provide reliable molecular evidence for the underlying growth-promoting mechanisms by integrating multi-omics strategies with phenotypic analysis. Plant–microbe interactions are complex and multifaceted, with core mechanisms involving gene regulation, metabolic exchange, signal transduction, and other biological layers. Benefiting from high-throughput analytical capacity, omics technologies offer powerful tools for dissecting these fundamental mechanisms. With the advancement of omics technologies, multi-omics integrated analysis has gradually become a core technical approach in studying plant–microbe interactions [23,24]. We hypothesize that *Streptomyces pactum* Act12 may influence root metabolic reprogramming, thereby modulating carbon partitioning and energy supply and potentially contributing to root developmental responses.

This study investigated the effects of Act12 inoculation on wheat growth, root development, gene expression, and metabolite accumulation using pot experiments combined with integrated transcriptomic and metabolomic analyses. It provides insights into the molecular basis potentially associated with Act12 mediated root developmental responses, thereby offering a theoretical foundation for the application of streptomycetes in sustainable green agriculture.

## 2. Results

### 2.1. Effects of Act12 Treatment on Wheat Growth and Root Development

**Figure 1:**
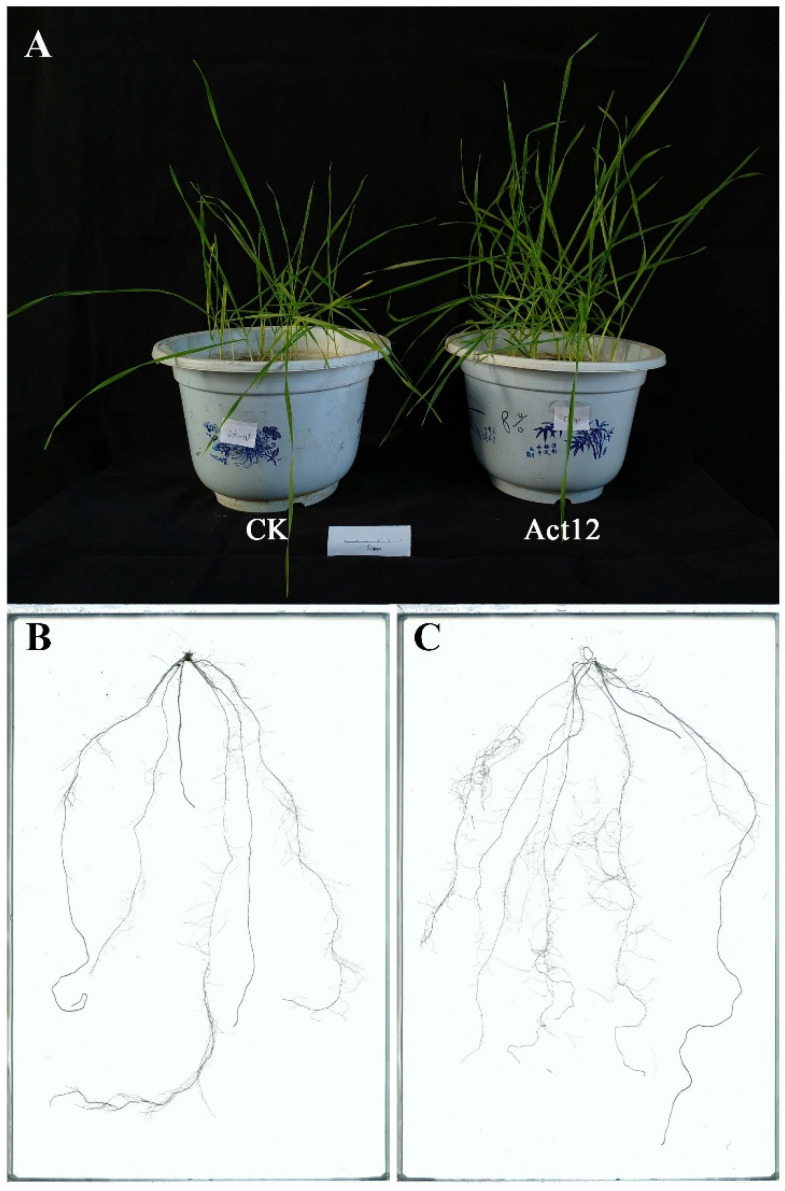
Effect of Act12 treatment on wheat growth and root system architecture. (**A**) Seedling phenotype in the CK and Act12-treated group. Scan of the root system in the CK (**B**) and Act12-treated group (**C**).

Act12 inoculation was associated with enhanced wheat growth (Figure 1A). Compared with the control, Act12-treated wheat showed 331.1% and 138.1% increases in fresh weight of aboveground parts and roots, respectively, with corresponding dry weight increases of 184.8% and 130.3%, respectively (Table S1). Root scanning analysis revealed that Act12 treatment significantly enhanced total root length, root surface area, root diameter, and root tip number by 44.6%, 73.3%, 34.0%, and 66.6%, respectively. This finding indicated that Act12 may contribute to enhanced root development (Figure 1B,C; Table S1).

### 2.2. Effects of Act12 Treatment on Gene Expression in Wheat Root Systems

To reveal the effect of Act12 inoculation on root system gene expression, the transcriptional profiles of the Act12-treated group and the control were compared and analyzed. The clean data for all samples ranged from 10.33 to 10.79 Gb, with GC content between 53.63% and 53.84%, and the Q30 base percentage exceeded 88.96% (Table S2). Mapping of the clean reads to the wheat reference genome yielded alignment efficiencies of 92.45–93.35%, confirming the high sequencing quality and data integrity of the transcriptome data. Principal component analysis (PCA) indicated that the repeated samples were well aggregated and that there was a significant distinction between the Act12-treated and control groups (Figure S1A).

**Figure 2:**
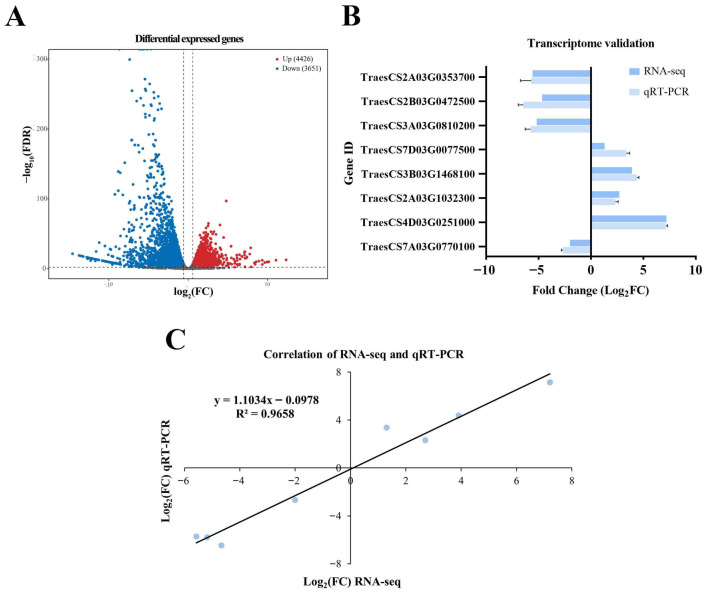
Differentially expressed genes identification. (**A**) The volcano plot of DEGs. (**B**) qRT-PCR validation of the transcriptome data. (**C**) The correlation between qRT-PCR and RNA-seq results.

A total of 59,250 genes were identified in the CK and Act12-treated groups. Based on |log_2_Fold Change| ≥ 1 and *p* < 0.01, 8077 differentially expressed genes (DEGs) were detected, including 4426 upregulated and 3651 downregulated genes (Figure 2A). To verify the reliability of the transcriptome data, eight DEGs were randomly selected for validation by qRT-PCR. All the primers used are listed in Table S3. The results showed that the qRT-PCR data were consistent with the RNA-seq analysis, with a linear correlation coefficient of 0.9658, thus confirming the reliability of the RNA-seq data (Figure 2B,C).

### 2.3. Classification and Enrichment Analysis of DEGs After Act12 Inoculation

**Figure 3:**
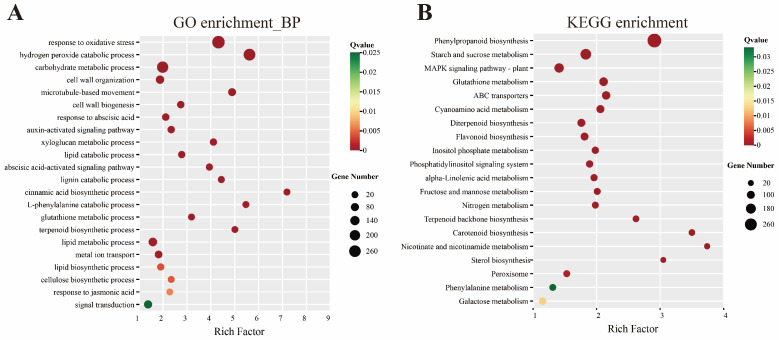
GO and KEGG analysis of DEGs. (**A**) GO enrichment of DEGs in BP ontology. (**B**) KEGG enrichment of DEGs.

GO classification of DEGs showed that cellular process and metabolic process were the main entries of the biological process (BP) ontology (Figure S2A), cellular anatomical entity was the largest entry in cellular component (CC) ontology (Figure S2B), and binding and catalytic activity were the main entries of the molecular function (MF) ontology (Figure S2C). The BP enrichment analysis showed that the DEGs were mainly enriched in the response to oxidative stress, hydrogen peroxide catabolic process, carbohydrate metabolic process, cell wall organization, microtubule-based movement, response to abscisic acid, auxin-activated signaling pathway, xyloglucan metabolic process, and terpenoid biosynthetic process (Figure 3A). These results indicated that Act12 inoculation was associated with genes related to antioxidant responses, energy metabolism, cell wall formation, hormone signal transduction, and secondary metabolite synthesis of wheat. These enrichments may reflect redox adjustment associated with beneficial plant–microbe interaction rather than detrimental oxidative stress.

The results of KEGG pathway enrichment analysis showed that DEGs were mainly enriched in phenylpropanoid biosynthesis, starch and sucrose metabolism, MAPK signaling pathway, glutathione metabolism, ABC transporters, diterpenoid biosynthesis, flavonoid biosynthesis, carotenoid biosynthesis, nicotinate and nicotinamide metabolism, and sterol biosynthesis (Figure 3B).

### 2.4. Characterization of Wheat Root Metabolome in Response to Act12 Inoculation

In order to investigate the effects of Act12 application on wheat root metabolites, an LC-MS/MS analysis was performed to qualitatively and quantitatively analyze root metabolites, and a total of 1361 metabolites were identified. PCA indicated that the repeated samples were well aggregated and that there was a significant distinction between the Act12-treated and control groups (Figure S1B). Following systematic classification and annotation of the aforementioned metabolites, terpenoids accounted for the highest proportion at 12.85%, followed by lipids (11.90%), flavonoids (9.05%), and sugars and alcohols (8.98%) (Figure S3A). KEGG classification revealed that the biosynthesis of other secondary metabolites category contained the highest number of metabolites, followed by carbohydrate metabolism and amino acid metabolism (Figure S3B).

**Figure 4:**
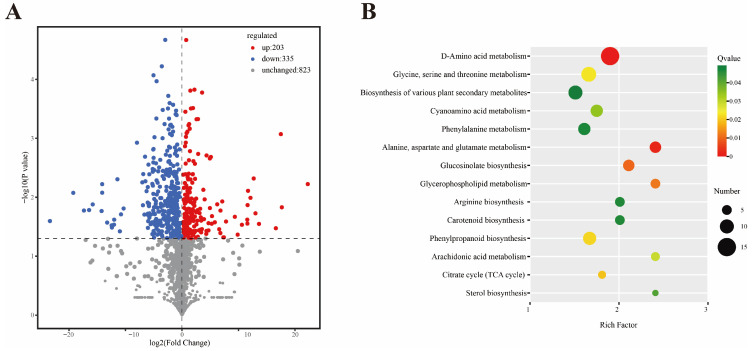
Identification and classification of DEMs after Act12 inoculation. (**A**) Volcano plot of DEMs. (**B**) KEGG enrichment analysis of DEMs.

Differentially expressed metabolites (DEMs) were identified with thresholds of FC ≥ 1.5, VIP ≥ 1, and *p* < 0.05. A total of 538 DEMs were identified, including 203 upregulated and 335 downregulated metabolites (Figure 4A). KEGG pathway enrichment analysis showed that DEMs were mainly enriched in D-amino acid metabolism, biosynthesis of various plant secondary metabolites, cyanoamino acid metabolism, phenylalanine metabolism, glucosinolate biosynthesis, glycerophospholipid metabolism, carotenoid biosynthesis, phenylpropanoid biosynthesis, TCA cycle, and sterol biosynthesis (Figure 4B).

### 2.5. Integrative Analysis of the Transcriptome and Metabolome

**Figure 5:**
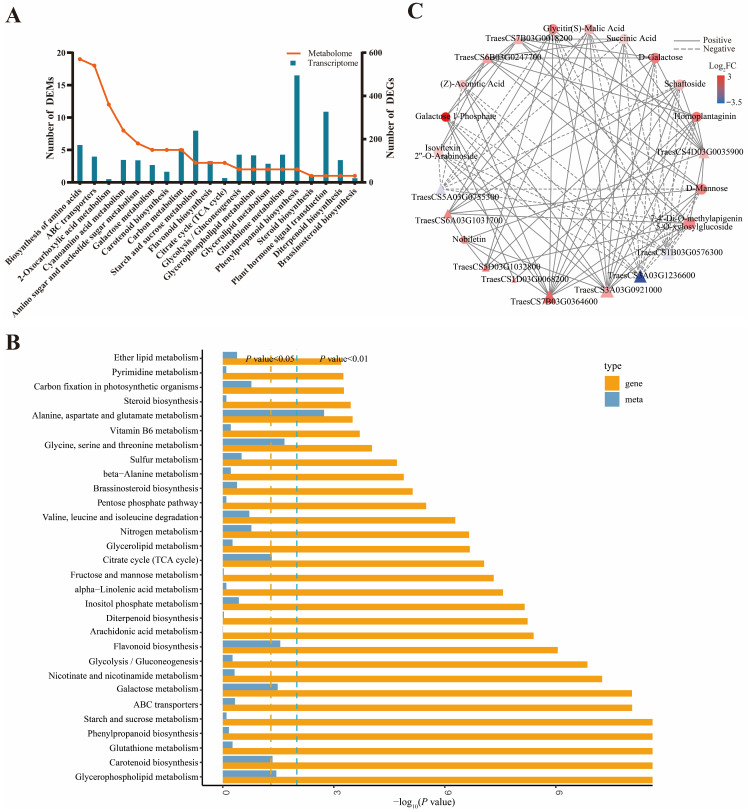
The identification of co-expressed KEGG pathways and the correlation of DEGs and DEMs under Act12 treatment. (**A**) The number of DEGs and DEMs associated with the top 20 co-expressed pathways. (**B**) Comprehensive analysis of transcriptome and metabolome data of the KEGG enrichment pathways. (**C**) Correlation network of key DEGs and DEMs. The nodes represent genes (triangles) and metabolites (circles), and the edges represent the correlation between them. The size of the nodes indicates the relative expression of the metabolites or genes. Solid lines denote positive correlations and dotted lines denote negative correlations.

To further explore the association between gene expression profiles and metabolite accumulation, we performed an integrated transcriptome and metabolome analysis. Through integrated KEGG enrichment analysis of all DEGs and DEMs, 81 enriched co-expressed pathways were identified (Table S4). The main enrichment pathways include biosynthesis of amino acids, ABC transporters, 2-oxocarboxylic acid metabolism, phenylpropanoid biosynthesis, galactose metabolism, carotenoid biosynthesis, carbon metabolism, and TCA cycle (Figure 5A). The KEGG enrichment histograms for DEGs and DEMs revealed significant enrichment in pathways including “Alanine, aspartate and glutamate metabolism”, “Glycine, serine, and threonine metabolism”, the citrate cycle (TCA cycle), flavonoid biosynthesis, galactose metabolism, carotenoid biosynthesis, and glycerophospholipid metabolism (*p* < 0.05) (Figure 5B). To investigate the relationship between DEGs and DEMs in wheat roots under Act12 treatment, co-expression network analysis was performed using thresholds of Pearson correlation coefficient (PCC) > 0.90 and *p* < 0.01 (Figure 5C). These results indicated that Act12 may participate in complex regulatory networks associated with amino acid metabolism, the tricarboxylic acid (TCA) cycle, galactose metabolism, carotenoid biosynthesis, glycerophospholipid metabolism, and sterol biosynthesis in wheat roots.

**Figure 6:**
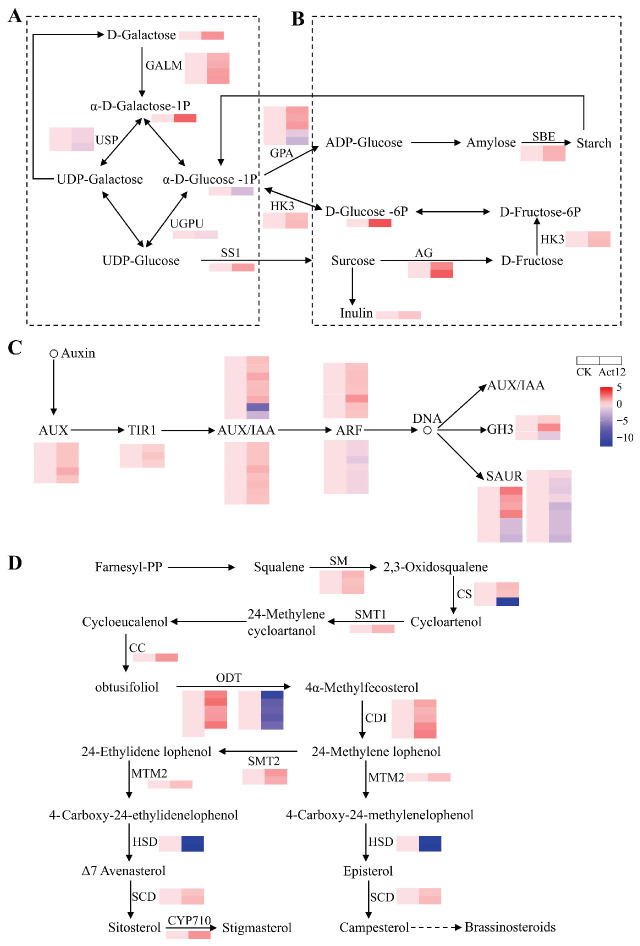
Changes in the main metabolic pathways under Act12 treatment. (**A**) Galactose metabolism pathway. (**B**) Starch and sucrose metabolism. (**C**) Auxin signaling pathway. (**D**) Sterol biosynthesis pathway. Solid arrows indicate direct relationships between metabolites, while dashed arrows represent in-direct associations.

To further explore the significantly enriched metabolic pathways identified above, we constructed targeted metabolic pathway diagrams based on the integrated transcriptomic and metabolomic analyses. These diagrams visually demonstrate the correlations and regulatory relationships between DEGs and DEMs in the core pathways after Act12 inoculation. In the galactose metabolism pathway (Figure 6A and Table S5), the levels of D-galactose and galactose 1-phosphate were significantly increased. All four genes encoding galactose mutarotase were significantly upregulated, whereas the three genes encoding UDP-sugar pyrophosphorylase and the single gene encoding UTP-glucose-1-phosphate uridylyltransferase were significantly downregulated. In the starch and sucrose metabolism pathway (Figure 6B and Table S5), the levels of D-glucose 6-phosphate and inulin were significantly increased. The expression levels of genes encoding starch branching enzyme and hexokinase-3 were significantly upregulated. In the auxin signaling pathway (Figure 6C and Table S6), the expression levels of 57 genes exhibited significant changes, of which 35 were significantly upregulated, and 22 were significantly downregulated. In the sterol biosynthesis pathway (Figure 6D and Table S7), the expression levels of 31 genes were significantly altered, with 23 significantly upregulated and 8 significantly downregulated. In the TCA cycle (Figure S4 and Table S8), the levels of aconitic acid, succinic acid, and malic acid were significantly increased. The expression levels of 19 genes exhibited significant changes, of which 17 were significantly upregulated, and 2 were significantly downregulated.

## 3. Discussion

The purpose of this study was to investigate the effects of Act12 inoculation on wheat growth, root development, gene expression, and metabolite accumulation, and to explore the molecular basis associated with Act12-mediated root developmental responses. Pot experiments showed that Act12 inoculation was associated with enhanced wheat growth. The integrated transcriptomic and metabolomic results suggested that Act12 treatment was associated with altered auxin signaling and coordinated changes in carbohydrate metabolism, the TCA cycle, and sterol biosynthesis pathway.

### 3.1. Effect of Act12 on Growth Promotion in Wheat

PGPR have been widely reported to promote plant growth via multiple direct and indirect mechanisms. The major direct mechanisms include modifying root system architecture to expand the absorptive surface area, improving mineral nutrient availability, and enhancing nutrient transport and utilization efficiency [25,26,27]. In this study, Act12 treatment significantly increased total root length, root diameter, and root tip number in wheat, thereby markedly expanding root surface area. An enlarged root surface area is generally associated with enhanced water and mineral nutrient uptake capacity, which in turn promotes aboveground growth. Previous studies have shown that Act12 can secrete IAA [28]. Concurrently, IAA functions as a pivotal phytohormone that regulates root system architecture. By modulating cell division and elongation, as well as lateral root primordium initiation, it promotes lateral root formation and elongation and further reshapes root structure [29,30]. On this basis, we propose that Act12 may promote wheat root development through auxin-related signaling modulation, potentially involving microbially derived IAA or other related signals, thereby contributing to improved nutrient uptake capacity and plant growth.

Beyond hormonal regulation, Act12 may also influence plant growth by modulating mineral element availability (especially iron) and host iron homeostasis. Previous studies have demonstrated that Act12 can produce siderophores, thereby potentially improving iron bioavailability in the rhizosphere [28]. In plants, intracellular iron transport relies on initial chelation with endogenous small molecules such as nicotianamine, followed by subsequent translocation via members of the nitrate/peptide transporter family (NPF) and vacuolar iron transporters (VIT) [31,32]. In this study, Act12 treatment significantly upregulated the expression of genes involved in iron homeostasis and transport in wheat roots, including nicotianamine synthase (NAS), as well as genes from the NPF and VIT families (see Table S9 for details). These results suggest that Act12 inoculation may be associated with improved iron bioavailability in the rhizosphere and with transcriptional responses related to host iron uptake and redistribution. However, because tissue iron concentrations were not measured, these observations should be interpreted as molecular signatures consistent with altered iron homeostasis rather than direct evidence of enhanced iron uptake.

In summary, the growth-promoting effect of Act12 on wheat may involve two major associated processes: (1) modulation of root system architecture, potentially linked to auxin-related signaling, resulting in increased root surface area and enhanced capacity for nutrient uptake; and (2) reshaping of iron bioavailability and host iron-homeostasis responses, potentially linked to bacterial siderophore production. These interpretations remain inferential and require direct physiological validation.

### 3.2. Effect of Act12 on the Auxin Signaling Pathway

Plant hormone signaling pathways constitute a key regulatory mechanism underlying plant growth promotion by PGPRs [33]. As the first identified plant hormone, auxin participates in nearly all aspects of the plant life cycle, including root and shoot morphogenesis, leaf and stem development, and gravitropic responses [34]. In this study, 57 DEGs were identified in the auxin signaling pathway after Act12 treatment. Notably, genes encoding the auxin transporter AUX1 and the receptor protein TIR1 were significantly upregulated (Table S6). AUX1 has been identified as a major auxin influx carrier. Loss of function of AUX1 impairs auxin polar transport, leading to defective root gravitropism and inhibited primary root elongation [35]. Given the reported IAA-producing capacity of Act12, bacterial IAA or other microbially induced auxin-related signals may influence auxin-associated transcriptional responses in wheat roots. Notably, Act12 treatment was not only associated with the auxin signaling pathway but also significantly influenced other hormone signaling pathways, such as the ABA pathway (Figure 3A). This suggests that Act12 may achieve precise control over growth and development by regulating the balance of multiple plant hormone signaling networks. However, since this study did not directly measure endogenous phytohormone levels (including IAA), further experiments are needed to confirm the direct association of Act12 with the auxin pathway.

### 3.3. Comprehensive Analysis of Transcriptome and Metabolome

In the present study, we performed an integrated transcriptomic and metabolomic analysis focusing on core metabolic pathways, including galactose metabolism, starch and sucrose metabolism, the TCA cycle, and sterol biosynthesis. Key regulatory genes and characteristic metabolites involved in these pathways were identified, revealing their potential synergistic regulatory network. Notably, transcriptomic and metabolomic responses were not always fully concordant across enriched pathways. For instance, phenylpropanoid biosynthesis showed strong transcriptomic enrichment but relatively few differential metabolites, which may partly reflect the limited coverage of widely targeted metabolomics and the complex regulation of metabolite accumulation.

Galactose, an abundant sugar component in plant root cell walls, exerts a direct influence on root development [36]. Research indicates that galacturonic acid, a derivative of the galactose metabolic pathway, directly regulates the polar growth of root hairs by participating in the formation of the polysaccharide network within the cell walls of root hair cells in *Arabidopsis* [37]. In this study, inoculation with Act12 significantly increased the levels of D-galactose and galactose 1-phosphate in the galactose metabolic pathway and upregulated the expression of the galactose mutarotase gene. This regulation may promote galactose activation, potentially contributing to the accumulation of metabolites such as galacturonic acid and facilitating root cell wall biosynthesis. Furthermore, intermediates of the galactose metabolic pathway, including D-glucose-1P and UDP-glucose, act as precursors for starch and sucrose synthesis. Among these, D-glucose-1P is converted into ADP-glucose (a key precursor for starch biosynthesis) by glucose-1-phosphate adenylyltransferase. UDP-glucose is converted to sucrose by sucrose synthase, which is specifically induced in root cells colonized by arbuscular mycorrhizal fungi, thereby modulating sucrose biosynthesis and allocation [38]. In our study, Act12 treatment significantly decreased D-glucose-1P content. Meanwhile, four genes encoding glucose-1-phosphate adenylyltransferase were significantly upregulated, whereas two were downregulated. Additionally, the gene encoding sucrose synthase was markedly upregulated. Furthermore, the level of D-glucose 6-phosphate, an intermediate in the sucrose-to-starch biosynthetic pathway, was also significantly increased. Starch and sucrose metabolism constitute the primary carbon storage and transport systems in plants [39]. Act12 may influence the synthesis ratio of these two compounds through carbon allocation mechanisms, thereby potentially regulating the efficiency of carbon storage and transport in plants.

The TCA cycle represents the primary pathway for meeting the energy demands of plants, thereby playing a crucial role in plant growth and development [40]. The breakdown products of galactose, starch, and sucrose yield glucose, which is catabolized via glycolysis to produce pyruvate. Pyruvate then enters the mitochondria and is converted to acetyl-CoA, which subsequently feeds into the TCA cycle. Our results show that Act12 treatment significantly increased the levels of key TCA cycle metabolites, including aconitic acid, succinic acid, and malic acid, indicating that Act12 may induce the active operation of the TCA cycle and enhance energy metabolism in root cells.

Sterols are essential lipid components of the plant plasma membrane and exert dual functions in plant growth and development. On the one hand, they modulate the physical properties of the plasma membrane to maintain cellular membrane integrity; on the other hand, they interact with membrane proteins and participate in vital physiological processes, including protein sorting, vesicle trafficking, and cell polarity establishment [41,42,43]. Sterol biosynthesis relies on energy supplied by the TCA cycle. Previous studies have shown that sterol content can regulate the polar growth of root hairs by affecting the localization and activity of membrane proteins [41]. In this study, Act12 treatment significantly altered the expression levels of 31 key genes involved in the sterol biosynthesis pathway, among which 23 genes were significantly upregulated. We speculate that Act12 may be associated with sterol biosynthesis-related transcriptional reprogramming, which could contribute to membrane organization and root developmental responses. In plant sterol biosynthesis, acetyl-CoA acts as the initial substrate of the mevalonate (MVA) pathway and provides the carbon skeleton for cycloartenol synthesis [44]. This pathway represents the core metabolic route for sterol production, converting simple carbon sources into complex plant sterols via a series of enzymatic reactions, and is therefore essential for plant growth and development [42].

Galactose metabolism, starch and sucrose metabolism, the TCA cycle, and sterol biosynthesis form a potentially tightly coupled metabolic network in plant roots. As key components of cell wall polysaccharides, galactose and its metabolites, including UDP-glucose and D-glucose-1-phosphate, can enter the starch and sucrose synthesis pathways and act as essential precursors for carbon storage and transport. Glucose derived from starch and sucrose degradation is converted to pyruvate via glycolysis and further processed into acetyl-CoA, which enters the mitochondria to fuel the TCA cycle and provide the energy basis for sterol synthesis. Acetyl-CoA also serves as the initial substrate for sterol biosynthesis, supplying the required carbon skeleton. Therefore, these four pathways may achieve coordinated regulation spanning from cell wall assembly to plasma membrane system maturation through carbon allocation and energy transfer.

In summary, Act12 may trigger metabolic reprogramming in roots by systematically regulating carbon flux and energy supply, thereby exerting comprehensive control over the root metabolic network. This may ultimately contribute to sterol biosynthesis, which could be associated with root developmental responses. However, the specific regulatory mechanisms underlying the effect of Act12 in this metabolic network remain to be further elucidated. Future studies may incorporate gene editing technologies to precisely regulate key genes within this network, along with field trials to assess their practical application performance. These efforts will help advance the practical use and implementation of Act12 in sustainable green agricultural systems.

## 4. Materials and Methods

### 4.1. Strain, Plant, and Growth Conditions

The PGPR strain *Streptomyces pactum* Act12 used in this study was provided by the Resource Biology Laboratory, Northwest A&F University. The concentration of the Act12 powder (prepared by solid-state fermentation) was 1 × 10^10^ CFU·g^−1^. The wheat variety used in this study was Jinmai 22, which is widely cultivated in northern China. The wheat seeds were disinfected with a 1% NaClO solution for 10 min, then rinsed three times with sterile water. The seeds were then germinated in a dark chamber maintained at 25 °C, with humidity regulated using damp filter paper. Three days after germination, seeds that had sprouted uniformly were selected and planted in pots (27 cm in diameter and 18 cm in height) at a density of nine plants per pot, with ten pots per treatment. Wheat seedlings in the treatment group were cultured in fine sand with 0.2% Act12 powder, while those in the control group were cultured in fine sand with 0.2% sterilized Act12 powder. Pots were watered with Hoagland nutrient solution every three days in a culture chamber maintained at 20 °C and 60% humidity, with a photoperiod of 12 h light/12 h dark, and cultivated until the four-leaf stage.

### 4.2. Determination of Wheat Biomass and Root System Architecture

The growth status of the wheat was recorded in both the control and treatment groups. Three pots were selected at random from each treatment as independent biological replicates (*n* = 3). From each pot, five seedlings were collected randomly, and the root systems were rinsed with sterile water to ensure that the roots remained intact. The wheat plants were then transected at the root-shoot junction and immediately weighed using an analytical balance to determine the fresh weight of both the aboveground and root systems. The wheat root systems were scanned using a root scanner (Microtek Scanmaker 800 plus, Microtek, Shanghai, China). The total length, surface area, diameter, and number of root tips of the wheat root were measured using a Win-RHIZO system (Regent Instruments Inc., Quebec, QC, Canada). The aboveground parts and root systems of wheat plants were then placed in an 80 °C oven to dry to constant weight. Subsequently, the samples were transferred to a desiccator to cool to room temperature, and then weighed immediately as dry weight.

### 4.3. RNA Sequencing and Data Processing

The root systems of the remaining wheat plants were collected and meticulously rinsed with sterile water to remove any impurities. The roots were then rapidly frozen in liquid nitrogen and stored at −80 °C for transcriptome and metabolome sequencing. Each treatment was performed in triplicate, with nine wheat root samples per replicate.

Total RNA was extracted from wheat roots using RNA prep Pure Plant Kit (Tiangen, Beijing, China) as per the manufacturer’s guidelines. The concentration and purity of the RNA were assessed by NanoDrop2000 (Thermo Fisher Scientific, Waltham, MA, USA). The RNA integrity was evaluated through agarose gel electrophoresis. RNA samples with high quality were selected for cDNA library construction.

cDNA library sequencing was performed on an Illumina NovaSeq platform (performed by Biomarker Technologies Corporation, Beijing, China) for the generation of 150 bp paired-end reads. Raw reads containing adapter bases or poly-N sequences and low-quality bases were removed. Subsequently, all the clean reads were aligned to the wheat genome: https://urgi.versailles.inrae.fr/download/iwgsc/IWGSC_RefSeq_Assemblies/v2.1/ (accessed on 17 January 2024). Differentially expressed genes (DEGs) were identified using DESeq2 R package [45] with an FDR-adjusted *p*-value < 0.01 and |log_2_Fold Change| ≥ 1. Gene Ontology (GO) [46] and Kyoto Encyclopedia of Genes and Genomes (KEGG) [47] databases were used for the analysis of DEGs.

### 4.4. Quantitative RT-PCR (qRT-PCR) Validation

To validate the sequencing quality of the transcriptome data, eight DEGs were randomly selected for examination by qRT-PCR. Primers were designed using the Primer Premier 6.0 software. The *TaEF* gene was used as a reference gene [48]. Total RNA was extracted as described above, and cDNA was synthesized by using FastKing RT SuperMix (Tian-gen KR118, Beijing, China). The gene expression analyses were performed by using the FastReal qPCR PreMix (Tian-gen FP217, Beijing, China) according to the manufacturer’s instructions. The PCR thermal cycling regime was 95 °C for 2 min, followed by 40 amplification cycles (95 °C for 5 s, and 60 °C for 15 s). The relative gene expression levels were calculated by the 2^−ΔΔCT^ method [49].

### 4.5. Metabolome Profiling by Widely Targeted Metabolomics

Wheat root metabolite analysis was conducted as previously described [50]. Frozen powders (50 mg) of root samples were transferred into 2 mL screw cap tubes containing 1000 μL extract solution (methanol:acetonitrile:water =1:2:1), and then incubated at −20 °C for 1 h. The samples were further centrifuged for 15 min at 12,000 rpm, 4 °C. 600 μL of the supernatants were filtered through a filter with a 0.22 μm membrane filter. The root extracts were then analyzed by widely targeted metabolomics using ultra-performance liquid chromatography-electrospray ionization-tandem mass spectrometry (UPLC-ESI–MS/MS; performed by Biomarker Technologies Corporation, Beijing, China). The Waters Acquity I-Class PLUS ultra-high performance liquid chromatography system was employed for chromatographic separation, equipped with a Waters HSS-T3 column (1.8 μm, 2.1 mm × 100 mm). The mobile phase comprised solvent A (pure water containing 0.1% formic acid and 5 mM ammonium acetate) and solvent B (acetonitrile containing 0.1% formic acid), with sample separation achieved via gradient programming. Chromatographic conditions were set as follows: flow rate 0.35 mL/min, column oven temperature 50 °C, injection volume 4 μL. The chromatographic eluate was alternately introduced into a triple quadrupole-linear ion trap mass spectrometer (QTRAP-MS) equipped with an electrospray ionisation (ESI) source for metabolite identification. Based on grouping information, the fold change for each compound was calculated, and the significance of differences was assessed using the two-tailed Student’s *t*-test. Orthogonal partial least squares discriminant analysis (OPLS-DA) models were constructed using the R language package. Model reliability was validated through 200 permutation tests, and variable importance in projection (VIP) values were calculated via multiple cross-validation. Selection criteria were set as *p* < 0.05, Fold Change (FC) ≥ 1.5, and VIP ≥ 1. KEGG pathway enrichment analysis was performed on the selected differentially expressed metabolites.

### 4.6. Integrative Analysis

Two-way orthogonal partial least squares (O2PLS) was employed to integrate transcriptomic and metabolomic data, assessing the intrinsic correlations between the two datasets. Through correlation analysis, functional annotation of genes and metabolites, and regulatory pathway analysis, the key pathways governing Act12-mediated root development in wheat were identified. The relationship between expression changes in pivotal genes and metabolites within these pathways was visualized in a heat map.

### 4.7. Statistical Analysis

In this study, physiological parameters were analysed using SPSS 27.0. Intergroup differences were assessed via one-way analysis of variance (ANOVA), with multiple comparisons conducted using the Least Significant Difference (LSD) test at *p* < 0.05. Visualisations were generated using GraphPad Prism 9 and Cytoscape software (version V3.9.1).

## 5. Conclusions

Collectively, Act12 inoculation was associated with enhanced root system architecture and substantial transcriptomic and metabolomic reprogramming in wheat roots, including pathways related to auxin signaling, carbohydrate metabolism, the TCA cycle, and sterol biosynthesis. Further targeted validation (e.g., hormone and sterol quantification, iron uptake assays, and colonization assessment) is required to establish causal links.

## Data Availability

Data are contained within the article and Supplementary Materials. The raw transcriptome data were deposited in the National Center for Biotechnology Information (NCBI) with the accession number PRJNA1426068.

## Supplementary Materials

The following supporting information can be downloaded at https://www.mdpi.com/article/10.3390/plants15101443/s1: Figure S1: Principal component analysis (PCA) of transcriptome and metabolome; Figure S2: GO classification of DEGs; Figure S3: Classification and annotation of the metabolites detected in the control and Act12 treatment groups; Figure S4: Changes in the TCA cycle under Act12 treatment; Table S1: Effects of Act12 on agronomic traits of wheat; Table S2: Summary of read statistics from RNA-Seq; Table S3: Genes used for qRT-PCR and their primer information; Table S4: Integrated analysis of KEGG enrichment pathways for transcriptome and metabolome data; Table S5: Differentially expressed genes and metabolites in the galactose metabolism, and starch and sucrose metabolism pathways under Act12 treatment; Table S6: Differentially expressed genes in the auxin signaling pathway under Act12 treatment; Table S7: Differentially expressed genes and metabolites in the sterol biosynthesis pathway under Act12 treatment; Table S8: Differentially expressed genes and metabolites in the TCA cycle under Act12 treatment; Table S9: Differentially expressed genes related to iron transport under Act12 treatment.
