# Supplementary material for: Integrative Transcriptomic and Metabolomic Analyses Provide Insights into the Regulatory Basis of Streptomyces pactum Act12-Associated Root Development in Wheat
Source: Plants (Basel). 2026 May 8;15(10):1443. doi: 10.3390/plants15101443 (PMC13210950; doi:10.3390/plants15101443)
Supplement: Supplementary file 1 [file plants-15-01443-s001.zip › Supporting Information/Figure S1-S4.pdf]

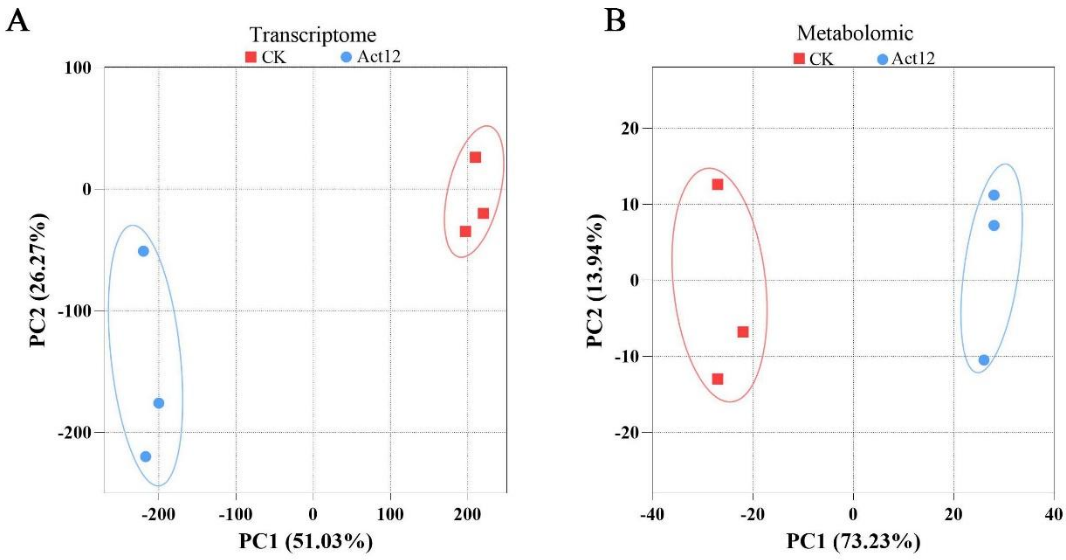

Figure S1. Principal component analysis (PCA) analysis of transcriptome (A) and metabolome (B).

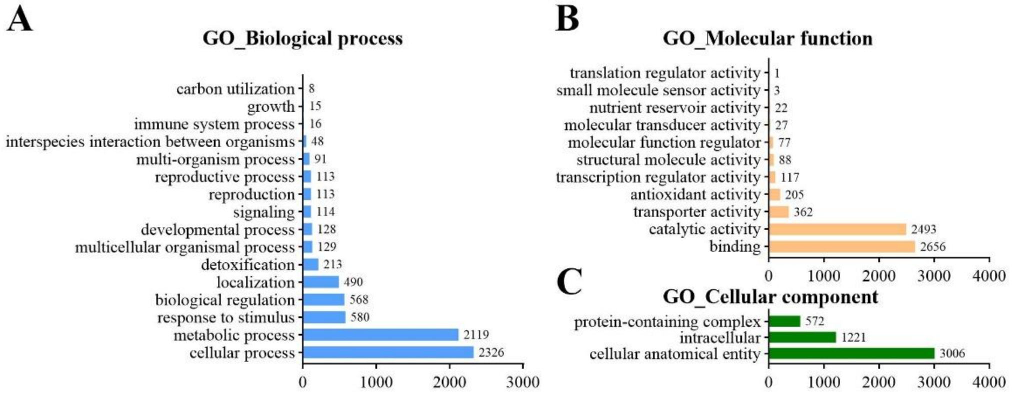

Figure S2. GO classification of DEGs. (A) Biological process ontology. (B) Molecular function ontology. (C) Cellular component ontology.

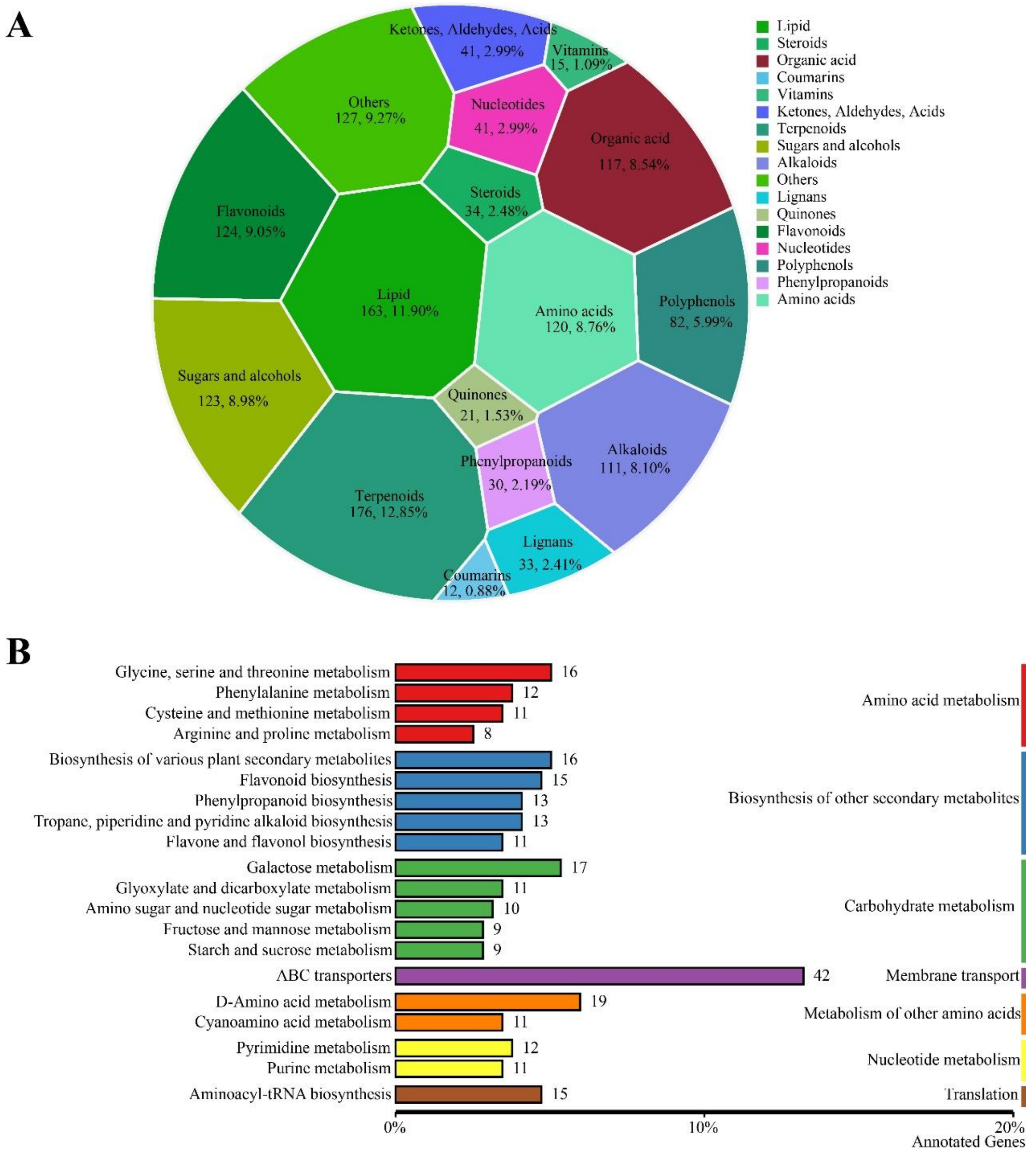

Figure S3. Classification and annotation of the metabolites detected in the control and Act12 treatment groups. (A) Distribution of classified metabolites. (B) KEGG classification.

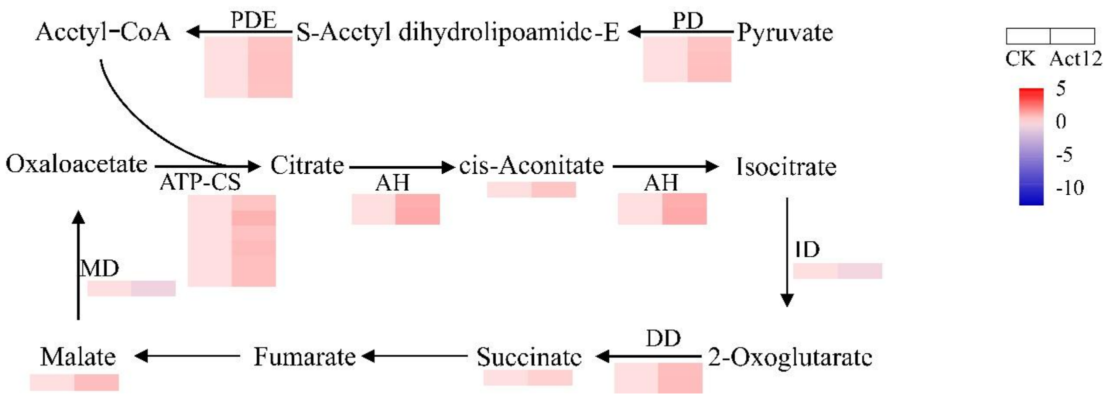

Figure S4. Changes in the TCA cycle under Act12 treatment.
